# Diabetic Retinopathy May Indicate an Increased Risk of Cardiovascular Disease in Patients With Type 1 Diabetes—A Nested Case-Control Study in Brazil

**DOI:** 10.3389/fendo.2019.00689

**Published:** 2019-10-11

**Authors:** Laura Gomes Nunes Melo, Paulo Henrique Morales, Karla Rezende Guerra Drummond, Deborah Conte Santos, Marcela Haas Pizarro, Bianca Senger Vasconcelos Barros, Tessa Cerqueria Lemos Mattos, André Araújo Pinheiro, Felipe Mallmann, Franz Schubert Lopes Leal, Luiza Harcar Muniz, Fernando Korn Malerbi, Marilia Brito Gomes

**Affiliations:** ^1^Department of Ophthalmology, State University of Rio de Janeiro (UERJ), Rio de Janeiro, Brazil; ^2^Department of Ophthalmology, Federal University of São Paulo, São Paulo, Brazil; ^3^Diabetes Unit, Department of Internal Medicine, State University of Rio de Janeiro (UERJ), Rio de Janeiro, Brazil; ^4^Department of Ophthalmology, Center for Endocrinology and Diabetes of the State of Bahia, Salvador, Brazil; ^5^Department of Ophthalmology, Regional Hospital of Taguatinga, Brasilia, Brazil; ^6^Department of Ophthalmology, Federal University of Rio Grande do Sul, Porto Alegre, Brazil; ^7^Department of Ophthalmology, University of Campinas, São Paulo, Brazil

**Keywords:** cardiovascular disease, diabetic retinopathy, diabetes, type 1, risk factors, microvascular

## Abstract

**Objective:** Cardiovascular disease, the leading cause of death worldwide, and diabetic retinopathy, the main cause of blindness in economically active populations, share clinical risk factors, and pathophysiological features. The aim of this study is to examine the association between diabetic retinopathy, cardiovascular disease, and common risk factors in patients with type 1 diabetes.

**Design and Methods:** This nested case-control study was performed in patients from the Brazilian Type 1 Diabetes Study Group, a nationwide survey that was conducted in Brazil and enrolled 1,760 patients with type 1 diabetes. A total of 342 patients were selected (57 cases with macrovascular disease and 285 controls who were matched for duration of diabetes and gender).

**Results:** In the exploratory analysis, stratified by cardiovascular disease, the following variables were statistically significant: age (*p*=0.037), hypertension (*p*=0.035), high BMI (*p* = 0.046), diabetic retinopathy (*p* = 0.003), and chronic kidney disease (*p* = 0.026). By multivariate logistic regression, patients with diabetic retinopathy were more likely to develop cardiovascular disease (OR 2.16, 95% CI 1.16–4.02, *p* = 0.015). Although to a lesser extent than diabetic retinopathy, higher BMI levels were also related to an increase in the risk of cardiovascular disease of 1.08 (95% CI 1.01–1.15, *p* = 0.024).

**Conclusion:** The presence of diabetic retinopathy indicates a greater risk for cardiovascular disease in Brazilian patients with type 1 diabetes. Further studies are warranted to determine whether a noninvasive exam, such as fundoscopy, could help identify patients who show an increased risk for cardiovascular disease.

## Introduction

According to the World Health Organization (WHO), cardiovascular disease (CVD) is the main cause of death worldwide, accounting for nearly 31% of all mortalities (1). Today, diabetes is the seventh leading cause of death globally and a major cause of blindness, chronic kidney disease, heart attacks, and stroke (2). In adults, diabetes, independent of other risk factors, confers an excess risk of CVD of approximately 2-fold, representing a macrovascular complication of diabetes and including coronary artery disease, peripheral arterial disease, and cerebrovascular disease (3, 4).

Hyperglycemia has injurious effects that are related to macrovascular complications (as reflected by CVD) and microvascular disease (diabetic retinopathy, neuropathy, and nephropathy). Intensive diabetes treatment reduces the risk of any cardiovascular disease event by 42%, compared with traditional therapy, as shown by the Diabetes Control and Complication Trial/Epidemiology of Diabetes Interventions and Complications (DCCT/EDIC), a study with 17 years of follow-up (5). In addition to hyperglycemia, many clinical risk factors are associated with CVD in patients with type 1 diabetes, such as hypertension, dyslipidemia, obesity, and diabetic kidney disease (1, 5, 6). Nonetheless, other risk factors that are related to lifestyle, such as smoking, unhealthy diet, stress, and physical inactivity, could also be important. All of these risk factors are associated with macrovascular and microvascular complications, such as diabetic retinopathy (DR) (6–8). Hyperglycemia is one of the most important risk factor for DR for both type 1 and type 2 diabetes. However, benefits of intensive glycemic control were more evident in type 1 patients (9). There are also, evidences of common genetic variance that increases the risk of DR associated with CVD (10, 11). Recent study in adolescents with type 1 diabetes and without DR showed that early signs of atherosclerosis in this population were associated with retinal microvascular changes, suggesting a common pathophysiology between the two conditions (12).

Diabetic retinopathy is the most common microvascular chronic complication and remains the leading cause of blindness in the working-age population (13). Due to the similarity in their risk factors and pathophysiology, involving inflammatory factors and endothelial damage, an association between DR and cardiovascular events is expected. Several studies have reported this link in type 1 and type 2 diabetes, as we can see in a meta- analysis published in 2011 (14). However, the number of such studies in type 1 diabetes is lower (15–18).

Despite the significance of CVD as the major cause of death and reduction in life expectancy in patients with type 1 diabetes, the routine screening of asymptomatic patients is not recommended according to the American Diabetes Association (ADA) statement. The advanced cardiac test such as: calcium score, pharmacologic stress echocardiography and exercise ECG testing with or without echocardiography are not cost-effective. Therefore, any benefit of non-invasive screening methods to identify patient subgroup at risk of CVD is interesting (19).

The aim of our study was to examine the association between DR with CVD and its risk factors in patients with type 1 diabetes.

## Materials and Methods

### Study Design

This case-control study included 342 patients with type 1 diabetes (57 cases and 285 controls with and without macrovascular disease, respectively), nested in the Brazilian Type 1 Diabetes Study Group (BrazDiab1SG). Detailed information on the main study has been published elsewhere (20). Briefly, this cross-sectional, multicenter study was conducted between August 2011 and August 2014 in 14 public clinics, comprising 1,760 patients who had been diagnosed by an endocrinologist, based on the typical clinical presentation.

A standardized questionnaire was administered, and clinical and demographic data were collected, such as age, gender, duration of diabetes, years of formal education, smoking (defined as the current use of 1 or more cigarettes per day), physical activity, alcohol consumption, self-reported ethnicity, diet, and hospitalization due to any cause in the past year. Economic status was stratified into high-, middle-, low-, and very-low-income, according to the Brazilian Economic Classification Criteria (21). The participants were also subjected to a clinical examination that included height (centimeters), weight (kilograms), BMI [weight (kg) divided by the square of the height (m^2^)], and blood pressure.

Data from medical records were also analyzed, and hypertension was defined, as reported by the patient, as at least 2 previous episodes of blood pressure ≥140/80 mm Hg, measured by a health professional. CVD was defined as positive information in the patient's medical records on 1 or more of the following conditions: coronary artery disease (angina, coronary artery bypass surgery, coronary angioplasty, myocardial infarction), peripheral vascular disease, and cerebrovascular disease.

Laboratory data were measured in a central laboratory, and the following ADA goals were adopted with regard to achieving adequate clinical and metabolic control (19): Good glycemic control was defined as HbA1c <7.5% for adolescents and <7% for adults. Poor glycemic control was defined as HbA1c ≥9%. HbA1c was measured by high-performance liquid chromatography (HPLC, Bio-Rad Laboratories, Hercules, California, US). For lipid management, we considered the following values as normal: triglycerides <150 mg/dL (1.7 mmol/L), HDL cholesterol >50 mg/dL (1.3 mmol/L) for women, and >40 mg/dL for men (1.1 mmol/L).

Renal function was assessed using the CKD-EPI equation (22) in adults and the Schwartz formula in adolescents (23) and was expressed as glomerular filtration rate (GFR) in milliliters per minute per 1.73 m^2^ (ml/min/1.73 m^2^).

Chronic kidney disease (CKD) was defined as the presence of a low estimated glomerular filtration rate (GFR <60 ml/min/1.73 m^2^) or elevated urinary albumin excretion (albuminuria ≥ 30 mg/dl). Creatinine was measured using a colorimetric assay kit (Biosystems).

Of the 1,760 patients from the original sample, 1,644 were screened for DR by mydriatic binocular indirect ophthalmoscopy by an ophthalmologist who specialized in the retina and had trained before the beginning of the study at an ophthalmological university center. DR was categorized per the international classification (24), but in this study we considered only the presence or absence of DR.

Of the 1,644 patients, 57 had macrovascular disease and were defined as “cases.” The group of cases was initially divided in the following duration of diabetes ranges: 0–5 years, 6–10 years, 11–15 years, 16–20 years, more than 20 years of disease. The proportion of gender in each range was analyzed. The 1,583 patients who did not present with cardiovascular disease, were random selected to match with the case group according to gender and duration of diabetes ranges. We set a ratio of 1 case to 5 controls, resulting in 285 controls. Ultimately, 342 patients, including cases and controls were analyzed.

The study protocol was approved by the coordinating center of the Pedro Ernesto University Hospital Ethics Committee (protocol: CEP/HUPE 2769/2010) and the ethics committee of each participating center. Informed consent forms were signed by the patient or his legal guardians. The research was conducted in accordance with the Helsinki Declaration.

The quality of the study was assessed using the checklist “Strengthening the Observational Report on Epidemiology” (STROBE) (25).

### Statistical Analysis

Initially, an exploratory analysis was performed to evaluate the relationship between CVD, DR, and certain clinical and social risk factors. For continuous variables, we performed the parametric test and those that did not present normal distribution were submitted to the Mann-Whitney test. Median and interquartile range [IQR] data were used for these variables. The variables with nonparametric distribution were: HbA1c, HbA1c mmol, triglycerides, HDL cholesterol, LDL cholesterol, and years of formal education. We performed the student's *t*-test and presented the data as standard deviation (SD) of the means for the others continuous variables that presented normal distribution. For categorical variables, we used chi-square test and presented the data as frequencies (percentage). We used Fisher's exact test for variables that presented cells with <5 elements, which occurred only in the smoking variable. Binary logistic regression was performed to assess the predisposing factors to CVD, considering variables with *p* < 0.2 in the univariate analysis and several clinically relevant variables. The following covariates were included: LDL cholesterol, hypertension (yes/no), BMI, smoking status (yes/no), HbA1c, DR (present/absent), age, economic status, years of formal education, and self-reported ethnicity (white/not white).

Exploratory Forward-Wald stepwise logistic regression was performed to determine the variables that contributed more to the discrimination between groups. Nagelkerke R-squared values were also calculated. Odds ratios (ORs) with 95% confidence intervals (CIs) were calculated when indicated. Two-sided *p* < 0.05 was considered to be statistically significant.

All analyses were performed using SPSS, version 20 (SPSS, Inc., Chicago, IL, US).

## Results

### Overview of the Study Population

Of the 1,644 patients, 57 presented with macrovascular disease and were considered “cases,” constituting 3.46% of all patients. The 285 controls were matched for gender and duration of diabetes. The clinical and demographic characteristics of the study population are shown in Table 1.

**Table 1:** Demographic and clinical data of the study population.

**Variable**	
*N*	1,644
**Demographic Characteristics**	
Gender, female, *n* (%)	917 (55.8)
Age, years	30.1 ± 12.0
Age of diagnosis, years	14.6 ± 8.9
Duration of diabetes, years	15.3 ± 9.3
Years of formal education, years	12.3 ± 3.8
**Economic status**, ***n*** **(%)**	
High	49 (3.0)
Middle	745 (45.3)
Low	795 (48.4)
Very low	55 (3.3)
**Insulin regimens**, ***n*** **(%)**	
Intermediate or long acting	80 (4.9)
Intermediate/long plus short acting	1.510 (91.8)
CSII	54 (3.3)
**Clinical Data**	
HbA1c (%)	9.0 ± 2.1
HbA1c, mean (SD), mmol/mol	74.5 ± 23.1
Use of angiotensin-converting enzyme (ACE) inhibitor, yes, *n* (%)	356 (20.2)
Use of Statin, yes *n* (%)	382 (21.7)
BMI, mean (SD), kg/m^2^	24.2 ± 4.2
Arterial hypertension, yes, *n* (%)	288 (17.5)
Macrovascular disease, yes *n* (%)	57 (3.5)
Current smoker, yes, *n* (%)	86 (5.6)
Chronic kidney disease, yes, *n* (%)	404 (24.6)
**Diabetic retinopathy, yes**, ***n*** **(%)**	589 (35.8)

*Data are presented as numbers (percentages) or means ± SD (standard deviation)*.

*BMI, body mass index; HbA1c, glycated hemoglobin; LDL-c, low density lipoprotein cholesterol; HDL, high density lipoprotein cholesterol; CSII, Continuous Subcutaneos Insulin Infusion*.

### Overview of the Study Population Stratified by Cardiovascular Disease (CVD)

In the exploratory analysis, patients with CVD were older (*p* = 0.037); had a greater prevalence of hypertension (*p* = 0.035), DR (*p* = 0.003), and CKD (*p* = 0.026); had a higher BMI (*p* = 0.046) and were more likely to be users of statins compared with patients without CVD. CVD was not associated in the exploratory analysis with years of formal education, economic status, self-reported ethnicity, use of angiotensin-converting enzyme (ACE) inhibitor, HbA1c, triglyceride levels, HDL cholesterol, LDL cholesterol, or smoking status. The results are shown in Table 2.

**Table 2:** Demographic, clinical and laboratory data stratified by Cardiovascular disease.

	**Cardiovascular disease**		
**Variables**	**Present**	**Absent**	***p*-value**
*N* (%)	57 (16.7)	285 (83.3)	
**Demographic Data**			
Gender, female, *n* (%)	26 (45.6)	130 (45.6)	1.0
Age, mean (SD), years	38.91± 15.01	34.42 ± 12.41	0.037
Duration of diabetes, mean (SD), years	22.59 ± 12.90	20.56 ± 10.31	0.27
Years of formal education, mean (SD), years	12.00 (3.00)	12.00 (4.00)	0.85
**Economic status**, ***n*** **(%)**			
High	1 (1.8)	8 (2.8)	0.28
Middle	22 (38.6)	146 (51.2)	
Low	31(54.4)	122 (42.8)	
Very low	3 (5.3)	9 (3.2)	
**Clinical Data**			
HbA1c mg/dl (%)	8.25 (2.62)	8.2 (2.30)	0.64
HbA1c, mean (SD), mmol/mol	66.66 (28.69)	66.12 (25.14)	0.64
Hypertension, yes, *n* (%)	24 (42.1)	80 (28.1)	0.035
Triglycerides, mean (SD), mg/dL	92 (59.75)	85 (58)	0.48
HDL cholesterol, mean (SD), mg/dL	49.85 (25.85)	54.70 (20.25)	0.18
LDL cholesterol, mean (SD), md/dL	99.40 (45)	101.20 (41.40)	0.16
BMI, mean (SD), kg/m2	26.09± 5.23	24.59 ± 4.20	0.046
Diabetic retinopathy, yes *n* (%)	39 (68.4)	133 (46.7)	0.003
Current smoker, yes, *n* (%)	3 (5.3)	18 (6.3)	1.00
Chronic kidney disease, yes, *n* (%)	28 (50.9)	97 (35)	0.026
Use of angiotensin-converting enzyme (ACE) inhibitor, yes, *n* (%)	23 (65.7)	78 (69.6)	0.66
Use of Statin, yes *n* (%)	33 (57.9)	85 (29.8)	0.00
Color race, white, yes (%)	29 (50.9)	168 (58.9)	0.26

*The data are presented as numbers (percentages), means ± SD (standard deviation) or median [IQR] (interquartile range). The p-value compares differences between the groups using Student's t-test. BMI, body mass index; HbA1c, glycated hemoglobin; LDL-c, low density lipoprotein cholesterol; high density lipoprotein cholesterol*.

Multivariate logistic regression was performed to determine the effects of social and clinical risk factors for CVD, wherein patients with DR were 2.16 times (95% CI 1.16–4.02, *p*-0.015) more likely to present with CVD. BMI was also associated with 1.08-fold (95% CI 1.01–1.15, *p* = 0.024) greater odds of having CVD. The independent variable included in the final adjusted multivariate binomial logistic regression model, explained 66% (Nagelkerke R) of the variance for the presence of CVD. The results of crude odds ratio and unadjusted *p*-value are described in Table 3.

**Table 3:** Multivariate logistic regression of cardiovascular disease (Present vs. Absent).

	**Unadjusted**		**Model 1**		**Model 2**	
	**Odds ratio**	***p*-value**	**OR (CI 95%)**	***p*-value**	**OR (CI 95%)**	***p*-value**
Diabetic retinopathy, yes	1.813 (0.897–3.665)	0.097	2.349 (1.274–4.332)	0.006	2.163 (1.164–4.020)	0.015
BMI, kg/m2	1.097 (1.022–1.177)	0.01			1.078 (1.010–1.150)	0.024
Age, years	1.015 (0.986–1.044)	0.322				
Arterial hypertension, yes	0.933 (0.436–1.994)	0.857				
Years of formal education	1.051 (0.961–1.149)	0.276				
Current smoker	1.254 (0.338–4.652)	0.735				
LDL cholesterol, mean (SD), mg/dL	0.993 (0.984–1.002)	0.134				
Chronic kidney disease	0.621 (0.318–1.213)	0.163				
**Economic Status**, ***n*** **(%)**						
High	0.380 (0.022–6.434)	0.503				
Medium	0.638 (0.109–3.734)	0.618				
Low	1.236 (0.231–6.622)	0.804				
Color race, white, yes	1.635 (0.854–3.131)	0.138				

*Data are presented as: odds ratio and CI 95% (95% confidence interval). BMI, body mass index; Model 1: after adjustment for presence of diabetic retinopathy; BMI, age, arterial hypertension, years of formal education, current smoker, LDL cholesterol, chronic kidney disease, economic status and self-reported ethnicity did not persist in the model. Model 2: after adjustment for duration of DM + BMI, age, arterial hypertension, years of formal education, current smoker, LDL cholesterol, chronic kidney disease, economic status, and self-reported ethnicity did not persist in the model*.

## Discussion

Patients with DR have a greater likelihood of presenting with CVD, even after controlling for the main risk factors. In our sample, the overall prevalence of CVD was 3.46% which is consistent with a report of the Wisconsin Epidemiologic Study of Diabetic Retinopathy that have shown rates of CVD of between 1.0 and 3.1% (17). The International Diabetes Federation (IDF) showed a prevalence of CVD, in patients with type 1 diabetes, ranging from 2.6 to 16.2% in high income countries. However, there is a lack of data from low-income countries (26). Although CVD is still an important cause of mortality and reduced life expectancy in patients with type 1 diabetes, a decrease in mortality on this population has been related both with a decrease in late diabetes complications and cardiovascular disease (6, 27).

The prevalence of DR in our population was 35.7% as we described earlier (28). This result is consistent with worldwide prevalence DR. However, although DR is still the leading cause of visual impairment among patients with type 1 diabetes, a recent review suggested that there is a declining trend in the prevalence of DR due to public health efforts (9).

The association between DR and CVD can be explained by the similarity in risk factors between both complications and by the evidence that microvascular and macrovascular complications of diabetes share pathophysiological mechanisms that are related to hyperglycemia (5). Hyperglycemia activates several intracellular signaling pathways that lead to oxidative stress and the overproduction of inflammatory markers. These occurrences contribute to retinal endothelial dysfunction, increasing vascular permeability—the chief event in the development of DR (29). There is evidence that tight glycemic control tends to be more benefit to prevent DR in patients with type 1 diabetes than in patients with type 2 diabetes, as shown in DCCT and in the United Kingdom Prospective Diabetes Study (UKPDS). The DCCT study, included only patients with type 1 diabetes and demonstrated in a mean follow up of 6.5 years, a reduced risk of 76% in DR and 54% in the progression of DR at the intensive glycemic control group (30).The UKPDS study, included patients with type 2 diabetes and the impact of tight glycemic control was a reduction in 25% the risk of microvascular endpoints, including the need of retinal photocoagulation (31).

There is also evidence of overactivation of the renin-angiotensin-aldosterone system as consequence of the hyperglycemia, impacting the pathogenesis of the microvascular complications in type 1 diabetes, affecting retina, nerves, and kidney (32).

The primary pathological mechanism in CVD is atherosclerosis, which is associated with endothelial injury and chronic inflammation (33).

CVD and DR share several risk factors, such as age, duration of diabetes, hypertension, higher BMI, and CKD. (6, 8) We also noted these variables in the descriptive analysis of this study and a separate report by our group that evaluated the risk factors that are associated with DR. (28).

Of these common risk factors, duration of diabetes could not be considered in our study, because it was used as a matching criterion in our population. Hypertension is another major risk factor for CVD and microvascular complications (5, 17). Hypertension has a direct correlation with CVD, because it is linked to arterial stiffness and thickness (6). Several studies, such as DCCT/EDIC, have shown that high levels of arterial pressure are strongly positively associated with the progression of DR (34). CKD, a result of diabetic microangiopathy, is also related to CVD, due primarily to its association with high fibrinogen levels, blood viscosity, lipoprotein levels, and platelet aggregation (17).

In our study, in addition to DR, BMI was a significant risk factor for CVD. Our population had a high prevalence of metabolic syndrome (35). These patients presented with clinical and laboratory indications that have been suggested to increase the risk for non-alcoholic fatty liver disease (NAFLD), which has also been associated with the development of CVD. There is evidence that obesity, an established risk factor for CVD, has a significant function in the endothelial damage in DR (36). Adipose tissue releases proinflammatory cytokines that induce insulin resistance and endothelial dysfunction, leading to diabetic angiopathy (37, 38).

We did not find association between smoking and CVD in our exploratory analyze. However, our sample included only a small number of smokers in cases (5.3%) and control (6.3%) groups and this might have influenced our results. This small number could be related to the fact that the percentage of adult smokers in Brazil has been significantly decreasing and according to the most recent data, it has a national average of 14.7% (39).

The strength of our study is that it included patients with type 1 diabetes which in general is not as studied as type 2 diabetes.

The study has some limitations. It was a cross-sectional study and so we cannot confirm causal associations between DR and its risk factors. Further, we evaluated DR by indirect ophthalmoscopy. Seven-field stereoscopic photography is considered the standard approach by the Early Treatment Diabetic Retinopathy Study (ETDRS) (40), but studies show significant agreement between the 2 methods (41). Also, retinopathy was not screened by the same physician, and for logistic reasons, inter-observer variability was not assessed. However, all exams were performed by an ophthalmologist who specialized in the retina and had been trained before the beginning of the study at an ophthalmologic university center. Further, our number of patients with CVD was small and data on CVD were based on medical records, no active screening was performed, according with ADA recommendations, that suggests systematic assessment of cardiovascular risk factors annually and does not suggest routine screening for coronary artery disease in asymptomatic patients with a high risk of CVD, such as our population (19). Nevertheless, the prevalence of CVD in our study was similar to that in other studies of patients with type 1 diabetes (17).

## Conclusion

The strong association between DR and CVD showed in our study suggests that presence of DR may be indicative of an increased risk for CVD. The presence of this ocular complication can identify those patients with type 1 diabetes who should be screened for CVD.

Cardiovascular disease is the leading cause of morbidity and mortality in patients with diabetes; thus, any effort toward its prevention is valuable. Fundoscopy could help to identify patients who are at higher risk of CVD. However, further studies are needed to understand the association between DR with CVD and the function of DR as a predictor of this significant and fatal comorbidity.

## Data Availability Statement

The raw data supporting the conclusions of this manuscript will be made available by the authors, without undue reservation, to any qualified researcher.

## Author Contributions

MG was the principal investigator of the study, designed the study and the survey questionnaire, supervised the project, and reviewed the manuscript. LGNM conducted statistical analysis, conducted the literature review, and drafted the manuscript. DS, MP, BB, and LHM helped to conduct statistical analysis and reviewed the manuscript. PM, KD, TM, AP, FM, FL, and FKM were responsible for collecting data on diabetic retinopathy in each center. All authors read and approved the final manuscript.

### Conflict of Interest

The authors declare that the research was conducted in the absence of any commercial or financial relationships that could be construed as a potential conflict of interest.
